# A Mixed-Methods Study of Creative Problem Solving and Psychosocial Safety Climate: Preparing Engineers for the Future of Work

**DOI:** 10.3389/fpsyg.2021.759226

**Published:** 2022-02-18

**Authors:** Michelle L. Oppert, Maureen F. Dollard, Vignesh R. Murugavel, Roni Reiter-Palmon, Alexander Reardon, David H. Cropley, Valerie O’Keeffe

**Affiliations:** ^1^Behaviour-Brain-Body Research Centre, Justice and Society, University of South Australia, Adelaide, SA, Australia; ^2^Psychosocial Safety Climate Global Observatory, Centre for Workplace Excellence, Justice and Society, University of South Australia, Adelaide, SA, Australia; ^3^UniSA STEM, University of South Australia, Adelaide, SA, Australia; ^4^Department of Psychology, University of Nebraska, Omaha, NE, United States; ^5^Australian Industrial Transformation Institute, College of Business, Government and Law, Flinders University, Adelaide, SA, Australia

**Keywords:** creativity, creative problem solving, engineers, exceptional cases, future of work, problem solving, psychosocial safety climate, teamwork

## Abstract

The future of work is forcing the world to adjust to a new paradigm of working. New skills will be required to create and adopt new technology and working methods. Additionally, cognitive skills, particularly *creative problem-solving*, will be highly sought after. The future of work paradigm has threatened many occupations but bolstered others such as engineering. Engineers must keep up to date with the technological and cognitive demands brought on by the future of work. Using an exploratory mixed-methods approach, our study sought to make sense of how engineers understand and use creative problem solving. We found significant associations between engineers’ implicit knowledge of creativity, exemplified creative problem solving, and the perceived value of creativity. We considered that the work environment is a potential facilitator of creative problem-solving. We used an innovative exceptional cases analysis and found that the highest functioning engineers in terms of knowledge, skills, and perceived value of creativity, also reported working in places that facilitate psychosocially safe environments to support creativity. We propose a new theoretical framework for a creative environment by integrating the Four Ps (Person, Process, Product, and Press) and psychosocial safety climate theory that management could apply to facilitate creative problem solving. Through the acquisition of knowledge to engage in creative problem solving as individuals or a team, a perception of value must be present to enforce the benefit of creativity to the engineering role. The future of work paradigm requires that organisations provide an environment, a psychosocially safe climate, for engineers to grow and hone their sought-after skills that artificial technologies cannot currently replace.

## Introduction

The future of work is characterised by the integration of humans and automation, artificial intelligence, and cyber-physical systems and is forcing the world to adjust to a new paradigm of working (Schwartz et al., 2019). Advanced digital technologies are now performing many routine and repetitive tasks previously undertaken by humans in a process known as digital transformation. Nevertheless, the kinds of tasks that digital systems can perform are limited by the inability of artificial intelligence to exhibit consciousness and original thought (Sheridan, 2019). Humans are likely to be in control of robots and other advanced technologies for the foreseeable future (Sheridan, 2019), and human factors experts assert that technology must now move to adapt to the human (De Winter and Hancock, 2021). As digital transformation leads to the increasing deployment of automation, artificial intelligence, and cyber-physical systems, the differences between human cognition and artificial cognition are becoming more clearly delineated. Higher-order elements of human cognition are becoming highly sought-after core skills precisely because artificial technologies cannot replicate them in the future of work paradigm. While the list of core skills in demand is long, a reoccurring point of interest is that of creativity and problem solving; Corazza (2019) reflects on human life in the cyber-physical society, reporting that psychosocial wellbeing in this new paradigm will depend on the development of human traits and abilities, particularly those related to creativity. *Creative problem-solving* includes the combination of skills such as communication, critical thinking, complex problem solving, and creativity. The focus of the future of work literature and the need for creative problem-solving in the workplace is not recent; the World Economic Forum [WEF] (2016, 2018, 2020) has been sharing these predictions for some time now. While the WEF and other organisations are focused on employees and the future of work, many other researchers and educators are heeding these predictions and changing student curricula to include inherently human skills such as creativity and complex problem solving (see Organisation for Economic Co-operation and Development[OECD], 2018, 2019; Cropley, 2020; Koshanova et al., 2021).

To meet the future of work demands, industry will need to employ creative problem solvers. At the same time, reports on the future of work identify professional occupations with a heavy emphasis on cognitive skills such as engineering, science, and health will be required. Engineering occupations make up 11% of the top 100 jobs least negatively impacted by digital transformation (Frey and Osborne, 2017). Since the future of work is promoting the need for creative individuals, and engineering is identified as an occupation of the future, we need a stronger understanding of how best to support and foster the environment that is conducive to creative problem solving/creativity to prepare engineers for the future. Moreover, it is recognised that we need to understand the workplace context (the psychosocial safety climate, Dollard and Bakker, 2010) that could boost creativity, exemplified through Reiter-Palmon and Kaufman’s (2018, p.192) quote: “*If creative people are put in situations that do not maximise their abilities, then the company loses many of the possible benefits.”*

**FIGURE 1:**
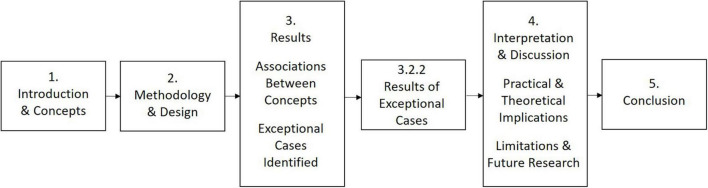
Flow chart of the present study.

Our innovative study employs mixed methods combining quantitative and qualitive perspectives to understand how creativity is experienced in the engineering workplace. Mixed methods research is well placed to answer questions that explore the concept of creativity, engineers’ experiences of creative problem-solving, and the psychosocial safety climate in the engineering workplace. In particular, this study interrogates exceptional cases to understand the features of engineers most prepared for the future of work. Figure 1 demonstrates the flow of the present study.

### Creativity and Engineering

Creativity is essentially any product or service that is new and effective. Creativity is defined as “…*the interaction among aptitude, process and the environment by which an individual or group produces a perceptible product that is both novel and useful as defined within in a social context”* (Plucker et al., 2004, p. 90). Zeng et al. (2010) state that a product or service can be regarded as creative when it is novel, appropriate, conquers a challenge, and – most important for engineers – satisfies a customer’s needs. The engineering profession is characterised as a trained, often accredited and registered, professional workforce employed in roles that allow, and expect, the production and development of innovations (Menzel et al., 2007). In engineering, creative problem solving involves cognitive-rich work such as divergent thinking, which is a core component of problem-solving (Cropley, 2015a). Divergent thinking is a reasoning process characterised by the ability to think flexibly, use imagination and remain original while making several alternative solutions possible from the information available (Guilford, 1957). In comparison, convergent thinking is a deductive process that considers facts and logic to arrive at the best solution (Cohen et al., 2013; Cropley and Cropley, 2015). This practical combination of convergent and divergent thinking are ingredients for successful creativity and innovation.

**FIGURE 2:**
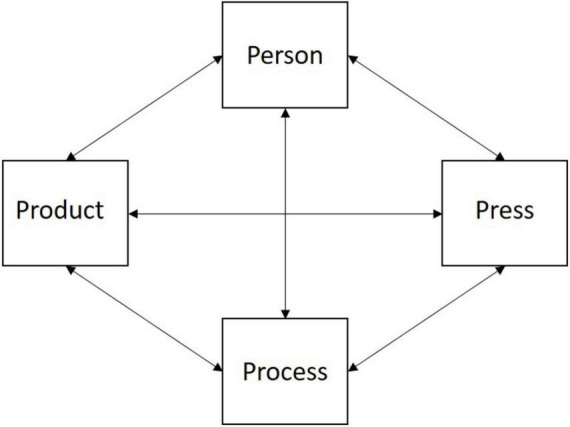
The Four Ps as a “System.” Items are adapted from “*Creativity in engineering. Novel solutions to complex problems*” by Cropley (2015a, p.12).

Paired with the definitions of creativity and convergent and divergent thinking, creativity in the workplace can be best understood through the lens of Rhodes’ (1961) Four Ps concept: Person, Process, Press, and Product. The *Person* is at the centre of the creative process in the workplace and includes psychological information internal to the individual with both stable (personality, intelligence, expertise) and malleable (attitudes, behaviour) aspects (see Zeng et al., 2010, p. 505). *Process* pertains to psychological information and includes perception, thinking and communication. *Press* is the work environment and includes the social climate (see Zeng et al., 2010, p. 505) and factors that can be motivating and conducive to *engaging* in creativity. *Product* is the outcome achieved through Person, Process and Press that is useful in the context of the workplace – particularly in engineering – and can manifest as a tangible product, process, or service. In the engineering domain, Cropley (2015a) presents the Four Ps as a “systems” concept of creativity adapted from Csikszentmihalyi’s (1998) systems perspective of creativity. In the context of a system, the Four Ps do not operate separately from one another but rather interact with each other (see Figure 2). The Four Ps framework is utilised to describe creativity that implies a uniform, stable set of conditions that favour the generation of novel and effective ideas, yet, it is more dynamic than static (Cropley and Oppert, 2018).

As previously stated, problem-solving – or the *Process* - is a core component of engineering work. Trevelyan’s (2014) research on expert engineers reports that engineering problems are rarely, if ever, written down, and what makes an expert engineer is years of experience, and part of that experience is the retention of knowledge. It has become evident that engineers are often taught using well-defined problems with a known solution during formal educational years. While many engineers do rely on known solutions to common, well-defined problems, most of these problems have already been solved (Trevelyan, 2014) and have well-defined solution processes. It is the new problems being faced by the future of work that do not have well-defined solutions; hence the demand for complex and creative problem-solving. Despite this, the ability to engage in creative problem solving is not an implicit skill or competency of all engineers: with many needing to be taught how to do this. However, the future of work requires more creative problem solving for more frequently encountered ill-defined problems, both technical or social. As Pretz et al. (2003) report, these types of problems typically lack a single, correct answer.

If the future of work calls for creative problem solving, then that ability has *value* for engineering firms creating an impetus to understand if engineers are creative. However, this information may be more implicit than explicit; Davies (2015, p.74) describes explicit knowledge as “*knowledge that the knower can make explicit by means of a verbal statement.”* Conversely, implicit knowledge is not easily verbalised, and tacit knowledge is not easily reported. Engineers may have better implicit – or tacit - knowledge of creativity than a self-report measure can tell us, and understanding this may be of use for preparations for the future of work, particularly when recruiting or re-training our current engineers. Equally, it is worthy to understand if engineers themselves perceive a value of creativity in their roles. While educators are aware of the new demands for skills in the future of work, there are engineers already practising and unlikely to return to the classroom. As such, this study asks:

RQ1: Do engineers possess implicit knowledge of creativity?

RQ1: Do engineers understand creativity in the context of problem-solving?

RQ3: Do engineers value creativity in their role?

### Psychosocial Safety Climate

At the same time that firms are seeking creative problem solvers, a concept gathering more attention is the positive impact on creativity when individuals are in a psychosocially safe work environment (see Gong et al., 2012; Lawrie et al., 2018). In these environments, workers are respectful and permissive of each other taking risks and expressing views (Edmondson, 1999). Recently, Greenbaum et al. (2020) identified the detriment on both psychological safety and creativity when the teams’ focus is on the “bottom line.” While a small number of researchers have identified a positive relationship between management and leadership in psychosocially safe workplaces and creativity of workers (see Carmeli et al., 2010, 2013; Reiter-Palmon and Royston, 2017), further exploration into the phenomenon of psychosocial safety and creativity is required.

Over time there has been a growth in literature and theory on the benefits of safe workplaces, starting with Safety Climate (Zohar, 1980), then Psychological Safety (Edmondson, 1999), and more recently Psychosocial Safety Climate (Hall et al., 2010). The Psychosocial Safety Climate (PSC) theory has built on the foundations of both Safety Climate and Psychological Safety literature (Dollard and Bakker, 2010). Now, a key component of workplace occupational health and safety, along with the concerns of the physical environment, PSC is focused on the psychological health of employees (Dollard and McTernan, 2011). Multilevel PSC expands on the Job Demands-Resource model proposed by Demerouti et al. (2001) by conceptualising the theory in terms of policies, practices and procedures in reference to workplace psychological health and safety. PSC is a characteristic of an organisation and therefore a target for controls to reduce work stress (Idris et al., 2012). The primary premise of PSC is that organisation managers and supervisors are the key enablers for a healthy psychological and social environment within their workplace. Psychosocial safety refers to freedom from psychosocial risks (such as work pressure, low decision authority). Over recent years, PSC has been tested in multiple domains and environments with many findings, including that poor PSC is associated with age-related cognitive decline (Wilton et al., 2019) and good PSC is associated with increasing workers’ initiative and workplace engagement (Lee and Idris, 2019). The main focus of PSC pertains to management support and commitment for the protection of worker psychological health and safety (Dollard and Bakker, 2010). The growing empirical evidence of the PSC makes it a potentially useful tool for establishing whether a safe psychosocial workplace climate could be positively associated with creativity in the engineering workplace. Throughout the literature, it has become clear that there is a gap in understanding how PSC is associated with creative problem-solving in the workplace. To address this gap, this study asks:

RQ4: How do engineers experience psychosocial safety at work?

RQ5: Is there an association between the psychosocial safety climate at work and creative problem-solving in the engineering workplace?

It is understood that assessing for creative potential alone is not enough and has been identified as a weak predictor of creative behaviour (Karwowski and Beghetto, 2019). As such, the current exploratory study aims to blend methods to understand how engineers experience creative problem solving and psychosocial safety in the engineering workplace. This research contributes to the work and organisational psychology literature by bringing the fields of creativity and psychosocial safety together to form new understandings in response to the future of work. Rea et al. (2017) emphasise the essential role of humans in the future of work, stating: *“The chain of discovery starts at the coalface with our human ability to notice the unusual or problematic – to swim through the stream of the unknowns and fuzzy concepts that cannot be fully articulated. This is where we collaborate to make sense of the world and create knowledge”* (p. 140). This statement illustrates the benefit of exploratory approaches to understanding the current construction of the future of work and the core human skills in demand. New insights can leverage the engineering workforce. The circumstances of the engineering profession (for example, profession protection in the future of work and the variety of tasks and roles conducted by engineers) provide a logical context for testing and exploring the theoretical propositions to identify implications for the engineering workforce and possible transferability to other domains.

## Methodology and Design

Building a research study that aims to understand the relationships between phenomena is complex. In light of limited existing research on associations between concepts included in this study makes it difficult to build on an established basis for hypothesis testing, experiments, or conducting and examining a case study. Thus, the following study is built on exploratory research methods and used mixed methods to provide a deeper, rich understanding of the potential association between the study phenomena. Exploratory research is undertaken when the phenomena under study have little systematic or empirical scrutiny, and the investigator wishes to inductively derive information to generalise about groups, processes, or situations related to the phenomena (see Stebbins, 2001). Exploratory research encourages the use of imagination, experience and insight, and can result in new and innovative ways to understand a phenomenon, while also allowing for rigour to attain validity and generalisability (see Reiter, 2013, Chapter 1).

Along with new insights derived from innovative approaches to understanding the phenomena, exploratory research can underpin further research that builds upon or tests initial findings. To strengthen this study, a cross-sectional design is used to enhance what generalisability there may be to the engineering workforce as a basis for further inquiry.

From an initial exploratory convergent design where the qualitative and quantitative data are collected concurrently and then merged together to provide comprehensive analysis (see Creswell and Creswell, 2018), the current study further evolved to include an extreme cases analysis of exceptionally positive cases. The purpose of analysing extreme cases is to highlight the most unusual variation in the phenomena under investigation, such as those that present maximum variation measured by different factors, or when outlier or opposite status is evident (Jahnukainen, 2010), as is the case in the current study.

A strength of qualitative research is its reflexivity, flexibility, and capacity to permit deep insight where quantitative research cannot. Thus, it is essential to harness these characteristics to explore not just the experiences of the “central themes” but investigate the characteristics and world-view of specific, well-performing individuals (Braun and Clarke, 2006; Richards and Hemphill, 2018). The narrower focus on exceptional data is an opportunity to extend the current understanding of these individuals, previously ignored and considered outliers, on the periphery of datasets (Seawright, 2016; Phoenix and Orr, 2017). The utility of this methodology has been realised by previous occupational research (Jónasdóttir et al., 2018) and is uniquely placed to extend our existing knowledge of creativity and psychosocially safe workplaces by exploring how the most creative engineers view their psychosocial environments (see Gong et al., 2012; Trevelyan, 2014; Greenbaum et al., 2020).

### Participants

The current study recruited a purposive, cross-sectional sample of 25 engineers (17 males, 8 females) from South Australia. Participants were recruited through social media posts, and a peak Australian engineering organisation advertised the study to its local seniors’ group members. All participants held a recognised engineering qualification, with at least one year of experience in the engineering workforce. The mean years of work experience for the sample was 16.33 years, and the mean age of the participants was 48 years. Ethics approval was obtained from the University’s Human Research Ethics Committee. Participants received written information about the research before data collection and provided signed informed consent. Participants completed the interviews and measures individually in private rooms on university campuses of convenience over seven months. There are two reasons that the number of participants equates to 25. First, 25 is the minimum number of participants for statistical analysis to be conducted. Second, through the interview process it was evident that data saturation was being reached as similar responses were accruing from individual participants, therefore making 25 the required number of participants for the present study to answer the research questions.

### Materials

#### Demographic Questionnaire

The demographic questionnaire collected information on participants’ age, education, employment, primary language, family, and hobbies.

#### Qualitative Data Collection

##### Semi-Structured Interview Questions

Semi-structured interviews were conducted to obtain meaningful data by exploring the phenomena and revealing participants’ knowledge and perceptions. Table 1 details the interview questions. To reduce the impact of social desirability, questions were designed to be non-directive and open (Grimm, 2010), eliciting knowledge, perceptions, understanding, and examples of creativity and creative problem-solving in the engineering workplace and how these relate to psychosocial safety. While plain language was used for clarity, further prompts were employed if participants did not understand the question. Interviews were conducted, audio-recorded, and transcribed verbatim. Care was taken to use plain English in the interviews due to the cultural and linguistic diversity within the sample. As such, the term “psychosocial” was not used in the interviews as it had the potential to confuse the participant’s interpretation of the question with discipline jargon. The participants were asked about psychosocial safety climate using the phrases “work health and safety” and “psychologically supported.”

**TABLE 1:** Set questions for interviews.

(1)	For a lot of engineers, a major process of their work is to solve problems and provide solutions. Can you tell me about a time when you had to engage in creative problem solving - where you had to come up with a novel solution to an issue or problem?
(2)	What are your thoughts on creativity in the engineering role?
(3)	We have many clear laws and procedures about work health and safety, particularly regarding your physical safety. From your perspective as an engineer, how have you felt supported psychologically in your workplace?

#### Quantitative Data Collection

##### Psychosocial Safety Climate 12 Item

The Psychosocial Safety Climate 12-item (PSC-12) scale (Hall et al., 2010) examines four core domains of PSC: management commitment, priority, communication, and participation for worker psychological health (see Table 2). PSC is a reliable (> 0.94) and validated measure. As a psychometric measure, PSC-12 was used to complement the interview question about psychological support in the workplace.

**TABLE 2:** Psychosocial safety climate 12 (PSC-12).

Domain		Items	
Management Commitment	(1) In my workplace senior management acts quickly to correct problem/issues that affect employees’ psychological health	(2) Senior management acts decisively when a concern of an employee’s psychological status is raised	(3) Senior management show support for stress prevention through involvement and commitment.
Priority	(4) Psychological wellbeing of staff is a priority for this organisation	(5) Senior management clearly considers the psychological health of employees to be of great importance	(6) Senior management considers employees psychological health to be as important a productivity
Communication	(7) There is a good communication here about psychological safety issues which affect me	(8) Information about my workplace psychological wellbeing is always brought to my attention by my manager/supervisor	(9) My contribution to resolving occupational health and safety concerns in the organisations are listened to
Participation and Involvement	(10) Participation and consultation in psychological health and safety occurs with employees, unions and health and safety representatives in my workplace	(11) Employees are encouraged to become involved in psychological health and safety matters	(12) In my organisation the prevention of stress involves all levels of the organisation

*Items are consolidated and reprinted from “Psychosocial Safety Climate: Development of the PSC-12” by G. Hall et al. (2010), International Journal of Stress Management, Volume 17(4), pages 382–383. Copyright 2010 by the American Psychological Association.*

The PSC-12 is scored from 12 to 60 and has established benchmarks: a score below 37.6 is poor with respondents at high risk of job strain and mental health issues, and a score above 41 is considered good, with scores in between deemed to be at moderate risk (Bailey et al., 2015). Based on the individual results, participants were allocated to one of three groups: (1) Low (Poor PSC), (2) Moderate (At Risk), and (3) Above Average (Good PSC).

### Qualitative Data Analytic Strategy

The research questions RQ1 through to RQ3 examine the participants’ implicit knowledge of creativity, if they understand creative problem solving (exemplified creative problem solving), and perceptions of creativity (value). To assess the phenomena, familiarity with the corpus of interview data was the priority. When deciding if the participants had implicit knowledge of creativity, an understanding was inferred by considering the gestalt of the interview responses together. Given that there are two questions about creativity, it allowed the participants to consider their experiences as the interviews progressed. At times, some participants stumbled on the first question inquiring about creative problem solving but later displayed knowledge when considering other questions and prompts. Additionally, the Four Ps (Person, Process, Product, and Press) and the definition of creativity were referred to by the authors when considering implicit knowledge and creative problem-solving.

At the outset of the study, the interview data were to be analysed using traditional qualitative analysis methods, such as Thematic Analysis. However, the information provided within the interviews presented an opportunity to quantify the data by identifying separate distinctions within participants’ degree of understanding. A scoring rubric was created with a three-point ordinal scale to indicate participants’ degree of (1) *Implicit knowledge of creativity*, (2) *Exemplified creative problem solving*, and (3) *Value of creativity* (see Table 3). Based on the responses from the individual participants, a score between 1 to 3 (1 = No, 2 = Somewhat, and 3 = Yes) was allocated for each query, including a rationale for that score.

**TABLE 3:** Scoring rubric for RQ1, RQ2, and RQ3 ranking and additional theme identification.

Question Number	Factor Being Explored	Question	3-Point Scale (1): No (2): Somewhat (3): Yes	Why?
1	Implicit Knowledge	Do they understand creativity?		
2	Exemplified Creative Problem Solving and Implicit Knowledge	Is their problem-solving example creative?		
3	Value of Creativity and Implicit Knowledge	Do they think creativity is important to the engineering role?		
		Other themes and points of interest		

This scoring rubric facilitates the interview data’s subjective rating and allows the individual assessors (MO and VM) to share and justify their decisions. The factors are deemed favourable and score highly if the participants:

1. Exemplify a high degree of understanding of creativity (implicit knowledge), for example, through direct and discrete statements that reveal comprehension through reiterating what creativity is, how it is used in their practice, or use of terms such as “tinker” or “jerry-rig” or “novel” relevant to the example provided. The overall examples provided also indicate the level of understanding; if their example is overtly creative, it is clear that the participant has a high degree of understanding creativity.
2. Exemplify creative problem solving through the description and articulation of the example provided by the participant, and whether it is a well-defined problem that was easily solved or one where the participant had to engage in creative problem-solving.
3. Believe creativity to be important (valuable) to the engineering role, which is expanded upon through direct and discrete statements

For example, a participant who scores a “3” on interrater rubric questions 1, 2, and 3, provides evidence that they understand creativity by providing examples of creativity, and at the same time acknowledge the importance of creativity to the discipline of engineering.

Once all interviews had scores allocated for each query, and themes or points of interest noted (in many instances, this includes notes on psychosocial factors), an independent academic with expertise on creativity (VM) analysed a random sample (*n* = 6) of interviews, allocating scores for each of the queries, and also reporting themes or points of interest. The purpose of including an additional rater was to ensure the primary author’s assessments were appropriate and unbiased. Through the questions we used to assess workplace PSC, creativity was also explored. After any discrepancies or biases were discussed in the interviews it was agreed that inter-rater saturation was met; both raters were independently scoring the interviews closely. As a result, a weighted Cohen’s Kappa for ordinal data was conducted, providing good interrater agreement (*K*_*w*_ = 0.61–0.80).

### Quantitative Data Analytic Strategy

#### Mantel-Haenszel Test of Trend

Quantifying the qualitative interview data provided three factors that could be assessed statistically, resulting in four factors for an association analysis: (1) Knowledge of Creativity (Implicit knowledge of creativity), (2) Value of Creativity, (3) Exemplified Creative Problem Solving, and (4) PSC-12. Before further qualitative analysis of the interview data to assess psychosocial safety at work, quantitative analyses were conducted to assess any potential associations between the four factors that could provide insights into all five research questions. We assessed implicit knowledge of creativity, exemplified creative problem solving, perceived value of creativity, and PSC-12 with a three-point ordinal scale from 1–3. Using SPSS statistical software, a Mantel-Haenszel Test of Trend was conducted on each potential association to search for associations between these factors.

The Mantel-Haenszel Test of Trend is suited to assessing the three-point ordinal ranked data, subject to data normality. The test is a non-parametric, conservative test reported to be more powerful than chi-square test for association with less sensitivity to small sample sizes (Agresti, 2007, Agresti, 2013). Consequently, the statistical significance result should be more accurate, making it particularly useful in smaller sample sizes (Laerd Statistics, 2016), as is the case with the current study. Requirements for the test include two ordinal variables and knowledge that the test assesses for presence of an association, but not whether the association is linear. While a statistically significant result may be present, that result may be curvilinear (Agresti, 2013; Laerd Statistics, 2016). The nature of the exploratory research design lacks dependent and independent variables, which suits the test, making the knowledge of an association useful for more in-depth analysis.

## Results

### Quantitative

#### Descriptive Measures

All the raw scores and subsequent results for each measure were normally distributed in the current study through visualisation of Q-Q plots. The PSC mean score was 41.40 (*SD* = 7.86). Table 4 shows the PSC-12 benchmarks, and Table 5 the count descriptive for each creativity factor.

**TABLE 4:** Psychosocial safety climate-12 benchmarks.

		PSC Benchmarks		
Group	*N*	Poor < 37.6	Moderate 37.7 – 40.9	Good > 41
All	25	7	2	16
Female	8	2	1	5
Male	17	5	1	11

**TABLE 5:** Counts for creativity factors.

Factor	Group	*N*			

Score			No (1)	Somewhat (2)	Yes (3)
Implicit Knowledge of Creativity	All	25	5	9	11
	Female	8	2	2	4
	Male	17	3	7	7
Exemplified Creative Problem Solving	All	25	4	9	12
	Female	8	2	1	5
	Male	17	2	8	7
Value of Creativity	All	25	3	11	11
	Female	8	2	1	5
	Male	17	1	10	6

RQ1 asked if engineers possess an implicit knowledge of creativity, with the results indicating that 44% (*n* = 11) of the participants were able to provide clear evidence of implicit knowledge of creativity.

RQ2 asked if engineers understand creativity in the context of problem-solving, with the results indicating that 48% (*n* = 12) of the participants were able to provide clear evidence of creative problem solving as exemplified in their descriptions.

RQ3 asked if engineers value creativity in their workplace, with the results indicating that 44% (*n* = 11) of the participants do value creativity in the engineering workplace.

#### Association Between Concepts

To further explore the data, a Mantel-Haenszel Test of Trend was conducted on the potential associations between the four factors. As noted above, the Mantel-Haenszel Test of Trend can inform a directional association but not the cause of the relationship; frequency scatterplot graphs generated during the analysis supported positive linear associations.

There was a statistically significant linear association between implicit knowledge of creativity and exemplified creative problem solving, χ^2^(1, *N* = 25) = 12.46, *p* ≤ 0.001, *r* = 0.721 (see Table 6). The relationship accounted for 52% of the variance. Implicit knowledge of creativity is positively associated with exemplified creative problem solving and vice-versa. We also found a statistically significant linear association between implicit knowledge of creativity and perceived value of creativity to the engineering role, and a statistically significant linear association between exemplified creative problem solving and perceived value of creativity to the engineering role (see Table 6 for all results). There were no significant relationships between the PSC-12 and the other factors.

**TABLE 6:** Mantel-Haenszel Test of Trend for liner associations between exemplified creative problem solving, implicit knowledge of creativity, value of creativity, and PSC-12.

Linear-by-Linear Association	Valid Cases (%)	*X* ^2^	df	Asymptotic Significant (2-sided)	Pearson Correlation	Significance (2-tailed)	*R*^2^ Linear
Exemplified Creative Problem Solving by Implicit Knowledge of Creativity	25 (100%)	12.462	1	0.000	0.721**	0.000**	0.519
Value of Creativity by Implicit Knowledge of Creativity	25 (100%)	11.879	1	0.001	0.704**	0.000**	0.495
Exemplified Creative Problem Solving by Value of Creativity	25 (100%)	4.619	1	0.032	0.439*	0.028*	0.192
Exemplified Creative Problem Solving by PSC-12	25 (100%)	0.000	1	1.000	0.007	0.927	0.542
Value of Creativity by PSC-12	25 (100%)	0.787	1	0.375	0.181	0.386	0.033
Implicit Knowledge of Creativity by PSC-12	25 (100%)	0.637	1	0.412	0.167	0.424	0.028

* Association is significant at the 0.05 level (2-tailed), ** Association is significant at the 0.01 level (2-tailed).

The statistical analysis of the quantitative data revealed curious information: while only three associations were found to be statistically significant, there was an overall pattern identified in the frequency scatterplots derived from the Mantel-Haenszel Test of Trend. A pattern of the same individual participants was repeatedly observed on the extreme positive results. Some cases scored positively on all factors, encouraging further examination of *why* this pattern was occurring. Jahnukainen (2010) describes the opposite status as a valid reason for conducting extreme cases analysis. We therefore analysed the extreme positive cases – *the Exceptional Cases* – to provide further insights into the study’s primary aim to understand how engineers experience creative problem solving and psychosocial safety in the engineering workplace while considering the demands of the future of work.

### Exceptional Cases

The outliers - or extreme cases - were examined, resulting in five cases being identified as exceptional cases and totalling 20% of the total participants. The Exceptional Cases comprise the following participants: 3C, 9I, 13M, 14N, and 25Y. The observed pattern of extreme cases in the Mantel-Haenszel Test of Trend frequency graphs was confirmed by conducting a count for each association of the six Mantel-Haenszel Test of Trend associations performed. The identified cases were plotted, summed, and simultaneously confirmed by referring to each participants’ case factor results. The extreme cases are termed *Exceptional Cases* based on the extreme positive locations on the frequency graph in the corresponding direction of the favourable responses. The identification of the Exceptional Cases and confirmation that the pattern of extreme positive disparity occurring for 20% of the sample warranted further analysis. The added steps of analysis embody the framework of exploratory methods where this study has developed further analysis as a result of *prior* analysis (see Greene et al., 1989).

#### Analysis of Exceptional Cases

To explore the Exceptional Cases, it is essential to return to the qualitative data to discover meaningful information pertaining to the quantitative data and reasons for Exceptional Cases in a purposive sample, which would not anticipate such extreme results. The initial analysis of the interviews aimed to collate the responses to the interview questions by quantifying the qualitative responses as ordinal data in earlier analyses, however, the overall gestalt of the Exceptional Cases is of more interest to revealing what is occurring in these cases. The qualitative analysis is useful for not only exploring the data but to answer research questions four and five:

RQ4: How do engineers experience psychosocial safety at work?

RQ5: Is there an association between the psychosocial safety climate and creative problem-solving in the engineering workplace?

An inductive approach of thematic analysis (Saldaña, 2009) was employed to analyse the qualitative data from only the extreme cases. Salient codes were identified through inductive – also known as *in vivo* – methods. Saldaña (2016) purports the *in vivo* method to be an ideal method when participant voices are featured; in other words, their *intonations* and use of language are essential in comprehending and representing the true meaning of what is being shared. Saldaña (2016) asserts the importance of differentiating codes and themes; a theme is an outcome of a code, and by repeatedly reading and reviewing the data, qualitative researchers cannot help but take note of themes. The three primary interview questions provided salient themes, which are identified through illustrative comments and interpretations. First, memos were created by the primary author in the documentation of the participants’ data which were subsequently revised and audited several times by the additional authors throughout the analytical and drafting stages to ensure the saliency of the themes

The Exceptional Cases were mostly heterogeneous with a mean age of 42 years and comprised two females and three males (see Table 7). Of the 19 potential factors from the demographic information and psychological measures in the collected data from the Exceptional Cases, six factors were commonly shared: (1) their highest qualification was a Bachelor’s degree; (2) no one had changed careers from engineering; (3) all scored above the low-risk benchmark for PSC; (4) all provided an implicit knowledge of creativity, and (5) all reported a perceived value of creativity in the engineering role (see Table 8). We note that a third of the total participants identified as female which is an over-representation of gender in the engineering profession in Australia, which presently sits at approximately 13 per cent (Kaspura, 2019). Overall, there were no meaningful differences found between male and female engineers anywhere in our study.

**TABLE 7:** Demographic information by exceptional cases.

Case	Gender	Age	Years of Engineering Experience	Specialisation	Country of Entry Qualification	Entry Qualification Obtained from	Highest Qualification	Changed from Engineering	Engaged in Further Education	Marital Status	Children	Grand-children	Primary Language
P3C	F	34	10–29	Chemical	Australia	University	Bachelors*	No*	Yes	Married	1	0	English
P9I	M	72	30 +	Electrical	England	Technical College	Bachelors*	No*	Yes	Married	2	3	English
P13M	M	27	<10	Chemical	Australia	University	Bachelors*	No*	Yes	Single	0	0	Greek
P14N	F	28	<10	Chemical	Australia	University	Bachelors*	No*	No	Single	0	0	Mandarin
P25Y	M	49	10–29	Mechanical	Australia	University	Bachelors*	No*	No	Married	2	0	English

* denotes commonality between all Exceptional Cases.

**TABLE 8:** Measures and Hobby information by exceptional cases.

Case	PSC Benchmark	Implicit Knowledge of Creativity	Value Creativity in Engineering	Exemplified Creative Problem Solving	Hobby	Hobby Description
P3C	Above*	Yes*	Yes*	Yes	Yes - 1-3 h per week	Dancing
P9I	Above*	Yes*	Yes*	Yes	Yes - 6-9 h per week	Amateur radio
P13M	Above*	Yes*	Yes*	Somewhat	Yes - 6-9 h per week	Bodybuilding
P14N	Above*	Yes*	Yes*	Yes	Yes - 1-3 h per week	Gardening and Playing guitar
P25Y	Above*	Yes*	Yes*	Yes	No	No hobby

* denotes commonality between all Exceptional Cases.

#### Qualitative Results of Exceptional Cases

Using inductive analysis of the qualitative data from the Exceptional Cases and interpretations of meaning in the data provided along with the commonalities noted, two primary themes were identified as: (1) *Rich knowledge and value of creativity*, and (2) *Management facilitation of psychosocially safe workplaces*, with an emergent theme of, (3) *Teamwork.* These are elaborated next.

##### Rich Knowledge and Value of Creativity

Exceptional Cases revealed a rich knowledge of, and value for, creativity in their roles. For example, P3C provided an example of explicitly valuing creativity in the engineering role but also implicit knowledge of creativity. P3C goes as far as to use the term *“novel”* and then second-guesses her use of the term, showing she does understand creativity, even if it is not an academic definition:

*I think creativity is thinking outside the box, what’s a novel, oh I shouldn’t say novel, but what’s another way to do it?” – P3C*

P3C further exemplifies this knowledge by describing a complicated situation involving an instrument malfunctioning in such a deviant manner that even the manufacturer had no precedent to guide solutions:

*“We came up with an interesting solution using one of our pumps and some flexible piping, a large number of connections, and we actually filled the system and managed to get a really dodgy, but effective, cleaning cycle on the dirty side to get it clean. We had to use a lot ‘what if we do this?’ and ‘what if we add this?’ and it worked!” – P3C*

P3C’s statement is interpreted as the process of divergent thinking that helps understanding, facilitating better outcomes than just the standard, well-defined solution. In other words, this could be considered innovation. P14N also describes a similar example of not simply selecting the known solution:

*“I’ve been pushing myself to do that [be creative], so I think the problem is when you know something then you probably know how to solve it, but if you go into a new problem you have never seen, you only have limited knowledge anyway, so if you do the same thing over and over again it might not be the best solution.” – P14N*

One of the most experienced engineers, P91, also shared similar thoughts on creative problem-solving in his role and provided an example whereby he, with his team, had to take some risks “*instead of taking the accepted method”* to find their solution. When asked if this was risky, P91 stated emphatically, “*absolutely.”* P91 suggests creativity as an engineer is:

*“Absolutely essential. You’ve got to think outside the box. You’ve got to. It’s the creativity that’s going to reduce the costs and shorten the time scale and meet what politicians and management want. You’re not going to do it [because] the management said we need it a year shorter*… *you can’t shorten the program by a year. If it’s only got to be this long, then what are you going to do about it? You’ve got to be creative. You’ve got to think of different ways of doing things. Think outside the box, work out how you’re going to do that. And that’s [sic] the creativity is essential.” – P9I*

For one participant, P13M, deviation in the direct work that he performs in his role as an engineer was not an option. P13M works in pharmaceutical production and, much like in other consumable production systems, the role does not lend itself to the direct, practical application of creative processes such as tinkering. However, he does not let that prohibit his understanding and application of creative problem-solving in his task despite there being few opportunities to do so. His approach illustrates that practical boundaries are not an excuse for not understanding, valuing, or implementing creativity in the engineering role. For example*:*

*“Boring means there are no deviations. Boring means there’s no production downtime. You know, you’re constantly pumping the product out and making money. So, in some cases, boring is good – not being creative is good. However, yeah, it’s crucial. It drives change. It brings us forward. I mean, it gives you a different approach to problem-solving. Continuous Improvement. It’s a good contributor to continuous improvement. I think it’s crucial [creativity], but unfortunately, most of us lack it” – P13M*

##### Management Facilitation of Psychosocially Safe Workplaces

When the participants were asked if they felt supported psychologically in their workplaces, the Exceptional Cases had much to share on being supported psychologically through either information and culture of their workplaces or direct experiences through their own needs. The questions in the interviews directly asked the engineers to describe a time that they had to engage in creative problem-solving. The Exceptional Cases did not mention management or stakeholders constraining their engagement in creative problem solving; rather, they expressed how they were encouraged and supported to be creative. This finding resulted in a theme of *management facilitation of psychosocially safe workplaces.*

Among good knowledge and experience of psychosocial safety and support in their workplaces, Exceptional Cases also revealed overt managerial support to engage in creative problem solving and provide the safety to test out their ideas. Exceptional Cases participants provided examples through their interviews, where evidence of open communication between management and multi-disciplinary team members facilitated effective solutions to problems. For example, P25Y is in middle management and experiences support from higher levels of management to take opportunities to test out ideas:

*“I love it [creativity]. Some people just don’t have enough! I love trying it! We are so fortunate where we’ve been given the opportunity to try stuff, pretty much to learn.” – P25Y*

*“I suppose, to encourage people to try even if it is a risk of failure so that you can learn.” – P25Y*

P14N, as an early career engineer, described how she relied on the knowledge and skills she was taught during her degree, and her manager suggested she try solving problems a different way. P14N’s experience, again, aligns with Trevelyan’s (2014) statements on engineering students being taught skills to solve problems that have known solutions, which is not always the reality when graduates move into applied work. For example:

*“When I was doing study, I was just ticking a lot of boxes. We were trained to think this way. When I joined here - my manager is a very creative person - and when I first get a problem, I was doing it a textbook way, putting the formula in there and then putting the variables in there, and then doing exactly what I was trained four years for, and then he [manager] inspired me to, ‘why don’t you just do something else?” – P14N*

The Exceptional Cases provided rich descriptive responses to the question about psychological safety and support in their workplaces. The two younger engineers (P13M and P14N) stated they felt supported but did not have any personal experiences where they required direct support. Nonetheless they perceived a high level of managerial engagement for those requiring support. The other three engineers had experiences of psychological safety and support they could elaborate on.

P3C shares that she has a mental health disorder and that she felt stigma due to the way some staff she had worked with in the past treated her, explaining that this carried through into her accepting work at her current place of employment, where psychological safety has improved:

*“When I first started working [as an engineer], I was always a little quiet about the fact that I have depression*… *I didn’t tell people about it because I didn’t want a bad view. And, truthfully, when it did come out, and it came out during a bad time in my life when I was working with [previous employer] as a contractor before I got my job [at current employer]. It was raised, ‘can you actually handle the stress?’ You know, because she’s got a mental problem? And I’m like, ‘yes’ but yeah, so it was, obviously, once known it came as part of how they viewed me.” – P3C*

P3C goes on to explain how she experiences her workplace and that despite an awkward initial conversation, their understanding of mental health has progressed:

*“But since then, you know, that was five years ago, and mental health has changed its stigma, and it is improved now, and I’m actually quite vocal, not that I have depression, but a mental health issue. Purely because I don’t want stigma to be there. I want people to know that yes, I have a mental health issue, but I’m totally capable to do my job! So, I think I try change the idea that people have of people with depression.” – P3C*

*[Investigator: And work is supportive?]*

“Yep! Truthfully, if I went to my boss and said, ‘I need a mental health day because I am not coping,’ he’s like *thumbs up*…*He’s good with that, and I think he, I think he also acknowledges that you can have those days.” – P3C*

The history of mental health and psychological support was expanded upon while interviewing P9I, who has over 30 years of experience as an engineer. He reports that it was well-managed before the 1980s. P9I reports:

*“Oddly enough, the whole person was considered well from when I was a younger engineer through ‘til I was in the 1980s, the early 80s period. The finance people got involved, and huge pressures were put on to meet time scale and costs and that sort of thing, and when that happened, the staff management went backwards, due to, I think, those financial pressures. We lost the ability to manage the whole person.” – P91*

Despite no longer being able to discuss family, personal, and psychological problems with staff; P91 highlights that with progression into management and more experience, instead of reprimanding staff who did not fit the environment, he would seek to find other means for improvement, such as moving them into a different team. P13M, a younger and less experienced engineer, shares his contrasting experience of psychological safety and support as a priority for his workplace:

*“If there is one thing that’s incredible about the larger corporation is the safety and psychological aspect. Everybody talks about safety and psychological aspect. Everybody talks about its safety. Everybody talks about reducing stress levels. It’s part of the culture. R U OK? That’s the biggest question.” – P13M*

*“They [management] try and encourage honest communication and obviously if you are experiencing an issue you are more than welcome to reach out to a manager or a colleague and report it, how you feel. They are very good like that.” – P13M*

Another young engineer, P14N, enthusiastically discussed psychological support from her workplace, particularly in reference to her gender and experience.

*“I have been [supported]. So, I can confidently answer that question. My manager has been supportive. I think I’m very lucky because you don’t normally get it.” -P14N*

As the only female in the company, P14N highlighted the positive impact of her manager supporting and prioritising her safety and boundaries. One example included an after-hours social event with a predominantly male group. Her manager spoke to her in advance and told her that if she feels uncomfortable or harassed, she should talk to him about it, and he will find a way to fight it and take the matter further if not addressed. She reports:

“I feel very supported. No such thing has ever happened to me, yet *laughs*, I hope not, but knowing that [support] is in the background is very good.” – P14N

Excessive job demands underpinned P25Y’s decision to change firms. He reports favourably on his current place of employment and describes it as *“pretty good.*” In his prior role, he said, “*my phone was never off. It was 24/7 for six years”* and described a physical reaction to the phone ringing, both at work and pervading his social life. P25Y currently works in a management position and, in contrast to his own previous experiences, he makes a point of checking that his staff are alright. He expressed the importance of awareness and how he had lost a young friend [not colleague] to suicide the week prior. He shared why he is aware and how he makes sure to keep an eye on his young male subordinates:

*“It’s probably just more the awareness of bullying. You know*… *you do of course see on TV and A Current Affair [TV news program] or whatever it is. You see scenarios of where people have been bullied in the workplace and self-harm. And, it’s more, I think, just more about looking after him.”*

“…*I hope the people in my department feel that they could just grab me and say, ‘hey do you have a sec?’ and we’ll go upstairs to the meeting room.” – P25Y*

*[Investigator: What about if you were the one?]*

*“I suppose I see the position I’m in. If I’ve got an issue, I need to make sure*… *I dragged my boss into a room the other week regarding safety, and, well, I nearly broke down at the time because I was just getting so frustrated about feeling that my team were getting put into unsafe situations, and it was like, ‘I’ve had enough!’ I suppose until you let fly and do get emotional*…*now I have seen a change in him.” – P25Y*

At first glance, the above description appears to criticise his current workplace. Rather, P25Y is stating that as a manager, if he needs to talk to his own managers and discuss his concerns, he can do this face-to-face because he feels comfortable doing so. In addition to emphasising the importance of managerial support, emerging from the current study is that teamwork is also an important factor for both creativity and psychosocial safety.

##### Teamwork

The influence of a positive psychosocial workplace was expected to relate to creative problem-solving in the present study, although not specifically explored. Nevertheless, teamwork emerged as a new theme in the rounds of revision of the data through direct examples of the use of *“we”* over “*I*.” In fact, in many examples, it was difficult to separate the impact of teamwork and creative problem-solving. The theme of teamwork and open communication between stakeholders in their work was evident in most of these exceptional cases, in particular, to facilitate creativity. P91 does not explicitly state the value of teamwork or examples of where the team or management helped, but he did use the pronoun *“we”* for all of the examples provided, even in his mentoring role at the end of his professional career; the results were because of “we” and not “me/I.” P3C was the same; she reports on an example of creative problem solving and illustrates that problem solving was a team pursuit when “*we came up with an interesting solution.”* These implicit examples of the problems being solved through a team process illustrate how an environment of teamwork and communication can lead to new and useful solutions. These examples demonstrate the safety afforded to them to try something, even if it fails, contributes to learning and is not seen as a waste. Here, P25Y also describes how teamwork and open discussions lead to creative problem solving by facilitating risk-taking:

*“Quite often in this meeting we talk, we’ll have our conditioning monitoring guys, we’ll have a couple of tradesmen, oil analysis guru, and we’ll be talking about an issue. And we’ll say, ‘what do you think the issue is? What are we going to do, and how can we make this better?’ And we might go, ‘Okay, well what about this?’ and at the end of the day, we will say, ‘let’s try it!’ I suppose, to encourage people to try even if it is a risk of failure so that you can learn.” – P25Y*

### Summary of Results

The purpose of this research was to answer the primary research question that aimed to understand how engineers experience creative problem solving and psychosocial safety. The current study employed a novel and rigorous approach with advanced iterative methodology and analyses through a mixed-method exploratory design which provided insights to answer specific research questions and provided both depth and breadth of the phenomena, leading to an understanding of the primary research aim. Quantifying the interview data with a second author experienced in scholarly research of creativity was useful as it provided agreement that could be assessed statistically. The final results and interpretations were also considered between the authors, and any misconceptions or disagreements were discussed and resulted in an agreement. The collaborative nature of involving academics from related specialised fields including creativity, human factors, and organisational psychology bolstered the overall interpretations and agreement of the current study. Data from the quantified interviews revealed significant positive associations between three factors: implicit knowledge of creativity, exemplified creative problem solving, and perceived value of creativity to the engineering role.

The first iteration of results in the current study led to identifying a clear contrast between specific participating engineers. As a group, the Exceptional Cases all provided detailed understanding and examples of both creativity and psychosocial safety in their engineering workplaces, with a strong perception of the value of creativity in the engineering role. While there was no statistically significant association between PSC-12 and other factors in the statistical analysis, the Exceptional Cases all provided above-average scores on the PSC-12 measure.

The next iteration of results led to further scrutiny and interpretation of the Exceptional Cases’ qualitative data. Transforming the qualitative data into ordinal ranked data gave insight into the expected responses of the Exceptional Cases in reference to creativity knowledge, examples, and value. Further examination of commonalities was conducted to explore if other demographic factors could explain the Exceptional Cases; however, nothing of salience was identified.

The final examination of the Exceptional Cases highlighted the following factors as meaningful to understanding the primary research question of how engineers experience psychosocial safety and creative problem-solving in the engineering workplace. This process answered RQ4, finding that engineers experience psychosocial safety at work, both formally and informally, with engineers generally experiencing good psychosocial safety. Engineers in more junior levels of experiences revealed their experience of psychosocial safety to be more formalised, whereas the more senior, experienced engineers felt their experiences were less formal but no less supportive. RQ5 asked if there is an association between the psychosocial safety climate and creative problem-solving in the engineering workplace? The deeper qualitative examination of the exceptional cases found that there is an association as the psychosocial safety climate and creative problem solving occurred through management facilitating safe environments to engage in creative behaviours. To summarise, the key factors that impact engineers experience of psychosocial safety climate and creative problem solving are (1) Knowledge and Value of Creativity, (2) Management Facilitation of Psychosocially Safe Workplaces, and (3) Teamwork.

## Interpretation and Discussion

### Knowledge and Value of Creativity

On the face of it, it makes sense that the factors (knowledge and value of creativity) would be associated; logic would imply that one cannot perceive the value of creativity if there is no knowledge of creativity because the value of knowledge derives from its *potential benefit* for the knowledge-holder (Pacharapha and Ractham, 2012). Furthermore, one may struggle to practise the cognitive task of creative problem solving without knowledge of creativity because knowledge is the ability to perform tasks and the ability to *use* the information (Watson, 1999). This recursive logic leads to the interpretation that if an engineer possesses knowledge of creativity, they will be able to exemplify it in their problem-solving pursuits, thus *adding to the perception* of value to creativity to their role. This finding echoes the recently formed theory of *Creative Behavior as Agentic Action* by Karwowski and Beghetto (2019) that individuals who transform creative potential into outcomes are informed by the individual’s creative confidence and perceived value of creativity. The perceived value of creativity is what sets the Exceptional Cases engineers apart; as Karwowski and Beghetto (2019) found, even those who have high creative potential and confidence to act creatively may not demonstrate creativity if they do not see the value in doing so. Researchers have identified that knowledge increases and evolves in specialisations – such as engineering - because individuals acquire “*know-what and know-how”* (De Toni et al., 2017). However, the source of the Exceptional Cases’ knowledge of creativity is unclear, which is unsurprising, and a reason for why *implicit*, and not *explicit*, knowledge was explored. Domain specificity, characterised as the ability to produce a creative output specific to that one domain (Cropley and Kaufman, 2019), while a plausible explanation, is unlikely to be related to the differences between the extreme cases in this study due to the purposiveness of the sample.

The importance of imparting knowledge in practice is a characteristic of engineering due to the evolving nature of the discipline through innovation and market demands. Due to the common practice of engineering educators teaching engineering solutions through well-defined problems, ill-defined problems that require divergent thinking are approached with less confidence. When an engineer is educated through well-defined problems, real-world, ill-defined problems provide a challenge that requires creative problem solving to provide solutions. For the engineer, creative problem solving can be experienced as *cyclical in nature*, as described by Reiter-Palmon and Murugavel (2020), where any difficulties encountered can result in returning to the original problem-solving process. However, for the engineer adept at creative problem solving, this can also be an opportunity to consider the problem in a new manner that results in a different formulation of the problem encountered (Reiter-Palmon and Murugavel, 2020), which was touched upon two of the Exceptional Cases.

Trevelyan (2014) asserts that it takes approximately ten years to be considered an expert engineer, and that process includes mentoring and the sharing of knowledge to maximise both professional and personal potential (Silva and Yarlagadda, 2014). The ability of an organisation to develop a competitive edge often resides in the knowledge bases and the abilities of individuals to solve problems creatively (Carmeli et al., 2013). To do so, engineering in the future of work must adapt and develop mechanisms that facilitate knowledge exchange in the form of mentoring and informal coaching as part of the work (Oppert and O’Keeffe, 2019), including open discussions and appropriate risk-taking to engage in creative problem-solving. Carmeli et al. (2013) report that developing workers’ capacity to creatively problem solve is a complex task and constitutes a major challenge for leadership in organisations.

### Management Facilitation of Psychosocially Safe Workplaces

The findings from the current study, bolstered with evidence from other research, highlight that for positive, effective outcomes in the workplace, including creative problem solving, the workers need to feel psychosocially safe. Workplaces with organisational climates that are psychosocially safe influence the work of engineers two-fold: first, the benefits of workplaces where management prioritises the communication, support, and inclusivity of workers in terms of psychological safety facilitates protection and investment in the team’s health and safety. Second, fostering a psychosocially safe workplace where management cultivates safety at the team level will foster an environment that encourages safe and open discussions, not just about psychological health and safety, but to also share and test out ideas without fear of failure. These findings are not altogether new; Carmeli et al. (2010) found that inclusive leadership, characterised by openness, accessibility, and availability, increases psychological safety, which, in turn, increases employee creativity. Learning from both failures and successes enables management and team members to continue creative problem solving and improve their practice as engineers. These benefits also extend to creating better products and solutions for their clients and stakeholders. The findings of the current study reveal that leaders, management, and the overall culture of the working environment for engineers needs to be enacted in practice and not just in policy and procedures.

The Exceptional Cases all reported above-average PSC-12 scores, indicating high levels of psychosocial support from management in all four dimensions. However, as a group, they experienced more practical requirements of psychosocial support, such as requesting time off for mental health care and engaging in open discussions about their psychosocial safety needs. Without further research with the current study participants, the extrapolated evidence is that those who require psychosocial support are in the best position to accurately assess if their workplace *does have* a positive psychosocial safety climate. There may be a disconnection between those who have relied upon management support and those who have not. Dollard and Bailey (2019) examined PSC-12 scores of 633 participants from 38 workgroups, finding a similar issue when looking at the range of scores and the standard deviations from each workgroup. The deeper analysis found that while some workgroups had high levels (above benchmark) of PSC, there were individuals in most of these groups providing the lowest possible PSC-12 score, indicating that those individuals need urgent attention (see Dollard and Bailey, 2019, p.419-420).

The current study explores the engineering profession but argues that psychosocially safe work environments that facilitate creative problem solving may benefit all organisations and workers. The engineering industry must continue to facilitate and support managers to psychosocially safe work environments to foster creativity. Such support provides safety for all team members to openly communicate, test out ideas, and engage in appropriate risk-taking, which, through a virtuous cycle, leads to more creative problem-solving. Recent organisational research acknowledges that psychosocial hazards and risks contribute to harm and are intrinsic to the design of work, but that they should be viewed as strategic points of prevention and/or intervention to reduce prevalent adverse outcomes such as work-related stress and psychological injury (Potter and O’Keeffe, 2020), which can, in turn, reduce the ability to safely engage in risk-taking and creative problem-solving processes. Organisations should pay careful attention to building a psychosocially safe environment in which there is deliberation, feedback exchange, critical reviews, expression of dissatisfaction, and suggestions to improve the current situation (Carmeli et al., 2013).

### Teamwork

Solving problems creatively in the workplace rarely occurs in isolation. In the engineering workplace, engineers have to coordinate with people and willingly and conscientiously contribute their expertise as it develops, so bi-directional learning is always a component of engineering practice (see Trevelyan, 2010). It is evident from the current study that in the engineering workplace, the extra ingredient to effective outcomes through creative problem solving is teamwork. Effective creative problem solving with teams is not separate from psychosocial safety at work. Psychosocially safe work environments facilitate the ability to speak openly and share ideas, feel safe to take risks without punitive measures (see Carmeli et al., 2013; Reiter-Palmon and Royston, 2017; Dollard et al., 2019; Edmondson, 2019). It also means being safe from psychosocial risks such as work pressure, low job control, low decision making latitude, and low work meaning (Dollard and Bakker, 2010). Group creativity has a spectrum of research findings, from being positive and conducive to producing novel ideas to actual decreases in creative production (see Coursey et al., 2018). Recent research has employed the methods of neuroimaging in an attempt to further understand creative problem-solving in teams (see Mayseless et al., 2019). The method by which teams find and comprehend a problem may be key to understanding and facilitating the collaboration of effective teamwork for creative problem-solving. Research by Reiter-Palmon and Murugavel (2018) has found that teams that engage in active understanding and discussion of the problem being faced generate more original ideas and experience higher satisfaction with less conflict. The overall findings from the (Mayseless et al., 2019, p.8) study concluded that in social contexts, cognitive control is an important factor in team interaction and creative team cooperation. They summarise with the notion that successful team cooperation that leads to creative ideas is cyclic, with a back-and-forth interaction of cognitive control and socio-emotional processes (such as empathy). The back-and-forth sharing of knowledge in both cognitive and team contexts is another finding that bolsters the concept of a psychosocially, open environment because this facilitates the spread and acquisition of new knowledge (De Toni et al., 2017).

Additionally, while the ability to teach engineers to think divergently in educational settings is being espoused, the engineers, as discussed, share information on the job, and if a group comprises some highly creative workers and some less creative, the knowledge exchange can still occur despite differing approaches leading to more possible complex outcomes (Coursey et al., 2018). Earlier research by Kennel et al. (2013) found that when some teams are tasked with finding the most appropriate and novel solution, their selection of the “best” solution may have been less accurate because they have less knowledge on quality and originality evaluations compared to that of expert assessors on these concepts. Again, the data arcs back to the first primary finding of the current study steeped in the value of possessing knowledge of creativity.

A potential point of difference between the Exceptional Cases and other engineers may be a bottom-line mentality, which focuses on one-dimensional thinking that revolves around the bottom-line (financial outcomes) while neglecting other competing priorities (Greenbaum et al., 2012). The focus on the inflexibility of costs is inevitable in most organisations, but specifically in engineering, where various external and internal stakeholders constrain project budgets and timelines. In the context of the current study, the bottom-line mentality has scope for serious consideration on why the Exceptional Cases were outstanding in their application and value of creativity in their engineering roles. Recent evidence suggests that the pursuit of bottom-line attainment is negatively related to *both* team psychological safety and creativity (Greenbaum et al., 2020). This is a potential workplace cultural factor to consider in light of the findings, with the Exceptional Cases exemplifying all of the factors favourable to creativity in the workplace, with examples of teamwork and positive psychosocial safety, but with little focus on financial restraints.

### Practical Implications

Engineering organisations and engineers need to possess both implicit and explicit knowledge of creativity to improve their creative problem-solving processes. However, it is not only the acquisition of knowledge of creativity that leads to effective creative problem-solving. For creative problem solving to occur in the engineering workplace, the perception of value also needs to be present. The inherent value, a confluence with knowledge, will enforce the benefit of creativity to their roles as engineers and influence their outcomes. The benefit of knowledge is *why* it is perceived as valuable (see Pacharapha and Ractham, 2012), thus placing the responsibility of imparting the value of creativity in the engineering domain on educators and experienced engineers to foster the value of knowledge and creativity through education and exemplification in practice.

**FIGURE 3:**
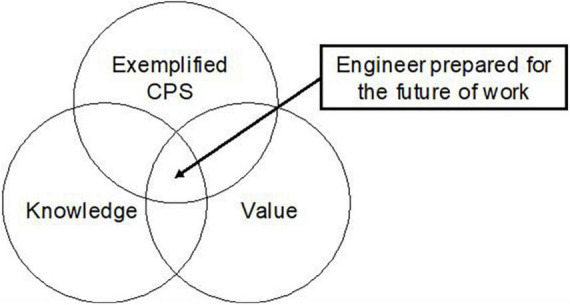
An engineer prepared for the future of work. Exemplified creative problem solving (CPS), Knowledge of creativity, and Value of creativity in the engineering role.

Workers of all disciplines will have to engage in continuous lifelong learning and development to meet the talent and skills requirements of the future of work (Volini et al., 2019; World Economic Forum [WEF], 2018). Investing in education and upskilling the current engineering workforce with knowledge of both creativity and how it can be harnessed to improve problem-solving is crucial for the engineering discipline to meet the demands set out in the future of work. The complex demands of today and the unpredictability of tomorrow requires more investment and support for human creativity (Pugsley and Acar, 2018). As such, it will be beneficial to have engineers that are representative of the Exceptional Cases group to meet those demands. All actors are required to be involved – organisations, stakeholders, educators, and engineers themselves – for effective uptake of knowledge acquisition of creativity and methods to engage in it. From this extensive exploration into factors relevant to the future of work, particularly for engineers, it is theorised that the engineer most equipped for the future of work will possess a high degree of knowledge and value of creativity with the ability to practise creative problem solving (see Figure 3).

This study’s findings recommend the application of the PSC-12 as a valid and reliable measure of an organisation’s PSC; however, those engineers relying on receiving practical psychosocial support from their managers when they require it are worthy of further research to identify what facilitating factors allow the workers to seek managerial support. If engineering firms are to use the PSC-12 to aggregate their firm’s overall PSC, they must also consider this in conjunction with a range of scores (Dollard and Bailey, 2019), paying particular attention to those with the lowest scores.

### Theoretical Implications

**FIGURE 4:**
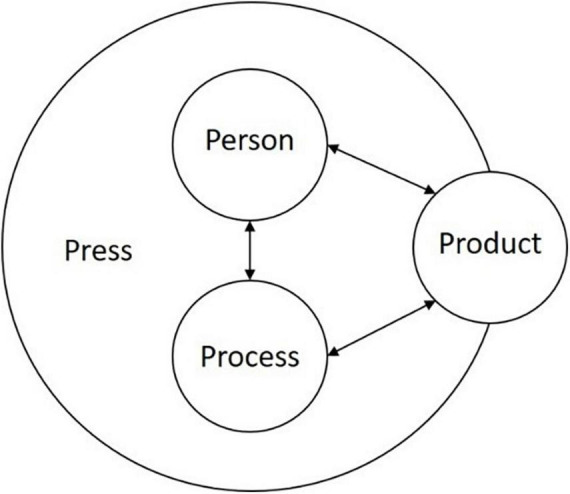
Theoretical framework of a Psychosocially Safe Engineering Environment that Facilitates Creative Problem Solving as envisaged through the Four Ps. Adapted from Rhodes (1961) and Cropley (2015a).

The future of work demands workers, not least engineers, who can effectively engage in creative problem solving to produce competitive and innovative solutions to problems. As a result of this study, we identify a theoretical framework to illustrate the engineer best prepared for the future of work through an environment where management facilitates creative problem solving. Much research has considered occupational factors that foster or impede creativity at work (see Carmeli et al., 2013; Tavares, 2016; Reiter-Palmon and Royston, 2017; Greenbaum et al., 2020) and some research has examined creativity and engineering (see Cropley, 2015b,^2016^). However, to the best of our knowledge, no research has examined the concept of creativity and PSC in the engineering workplace. Returning to the introduction of this study, the ideal framework to understand how engineers experience positive psychosocial safety and creative problem solving can be understood and visualised through Rhodes’ (1961) Four Ps (see Figure 1 above). We reconstruct the Four Ps to include the findings of the current study. The environment (Press) includes basic resources for engineers to complete their tasks, including appropriate plant and equipment and the added benefit of a psychosocially safe climate to engage in the tasks required to do their job. Within that environment (Press), the engineer (Person) is safe to communicate with one another and management about their ideas and work together in the environment (Press) to solve the problems. The problem solving (Process) occurs within the environment and can either happen within or through an individual worker or between team members. The process of creative problem solving facilitates engineers to create products and solutions (Product). Figure 4 illustrates the theoretical framework; the Product sits on the border of the Press because engineers’ creative problem solving does not just occur to solve stakeholder problems - it can extend into their own work environment to improve or personalise (see Zeng et al., 2010) their resources to meet their demands, manifesting as both systematic solutions, or practical solutions.

### Limitations and Future Research

The positivist view of psychology that relies on large sample sizes and statistically significant results can sometimes fail to capture the gestalt of the phenomenon being explored. An analysis of 25 participants should be interpreted with a degree of caution when using statistical analyses to establish statements of significance. For instance, the small number of participants may have made it difficult to find a signification association between PSC and the other creativity factors. While the findings strongly support the notion that engineers who possess knowledge and value of creativity in the engineering role are best placed to operationalise this at work, as with any correlation, causation cannot be inferred.

To reduce common source bias (ratings from one person), an author with expertise in their respective discipline of creativity was recruited to validate the interpretations of findings (see Favero and Bullock, 2015). In future research, expert raters of exemplified creative problem solving should be employed; however, real-time assessments of creative outcomes through observations or interventions would be beneficial to understanding the practical impact from multiple perspectives further afield from academics, including managers and stakeholders. The diversity of the participants is a strength; however, the findings may not be highly generalisable due to their purposiveness. Nonetheless, the findings could be further considered by Australian and other OECD engineering firms to understand the composition of factors impacted by enhancing the knowledge of creativity in firms with psychosocially safe organisational climates.

The future of work paradigm is augmenting the traditional understanding of teams as workers are becoming more distributed with the lessening reliance on the physical workplace, and remote working becoming a viable and attractive option for many workers. Through this lens, Schwartz et al. (2019) encourage organisations to reconsider how they foster both cultural and team connections in the future of work. In light of more remote working and interactions between multidisciplinary team members, psychosocial safety needs further investigation. The PSC-12 is a valid instrument for assessing organisational level PSC, however, further comprehension of the lived experience of engineers, and other workers, relying on practical psychosocial support needs to be examined. Interventions through education providers to provide professional development that promotes information on creative problem-solving mechanisms would provide opportunities for more evidence on the perceived value and the resulting outcomes.

Value needs further understanding in the creativity literature for scholars, educators, and external stakeholders to exemplify why investing in knowledge acquisition of creativity is beneficial in light of the future of work paradigm.

## Conclusion

The novelty of exploring the various factors through an exploratory study is a strength, as exploratory mixed-methods research is laborious and not afforded to many scholars. In fact, the approach to understanding the phenomena through exploratory methods epitomises *meta-creativity* (see Runco, 2015). By its very nature, exploratory research can balance the equal strengths and weaknesses found in its methods and discoveries. The study’s strengths include the explicit exploration into the engineering workforce, particularly if, as the future of work literature espouses, the discipline of engineering is protected in digital transformations. Engineers who possess implicit knowledge and value of creativity are able to engage in effective creative problem-solving. The engineers who can cater to these factors are best suited for what the future of work is currently demanding. Finally, it is not only the factors possessed by the engineer prepared for the future of work that will facilitate the effective transition to the new work paradigm. While the differences between human cognition and artificial cognition are becoming more clearly delineated, they also have to engage in more interactions in almost every work environment. Organisations must also provide a fertile environment – a psychosocially safe climate – for engineers to grow and hone their sought-after, core skills that, for the foreseeable future, cannot be replicated by artificial technologies in the future of work paradigm.

## Data Availability Statement

The datasets presented in this article are not readily available due to continued research on other aspects of the data. All quantitative data are provided in the manuscript. Requests to access the datasets should be directed to the corresponding author MO, michelle.oppert@unisa.edu.au.

## Ethics Statement

The studies involving human participants were reviewed and approved by University of South Australia Human Research Ethics Committee. The patients/participants provided their written informed consent to participate in this study.

## Author Contributions

MO conceptualized the design, collected and analyzed the data, interpreted the findings, and wrote the first draft and subsequent revisions of the manuscript. VM reviewed the interview data and provided inter-rate agreement. MD, RR-P, and DC provided methodological and theoretical guidance. VO’K reviewed the qualitative findings and provided agreement. MO, MD, AR, and VO’K contributed to writing the manuscript. All authors approved the revisions and submitted manuscript.

## Conflict of Interest

The authors declare that the research was conducted in the absence of any commercial or financial relationships that could be construed as a potential conflict of interest.

## Publisher’s Note

All claims expressed in this article are solely those of the authors and do not necessarily represent those of their affiliated organizations, or those of the publisher, the editors and the reviewers. Any product that may be evaluated in this article, or claim that may be made by its manufacturer, is not guaranteed or endorsed by the publisher.
